# Hemoglobin A1c for Diagnosis of Postpartum Abnormal Glucose Tolerance among Women with Gestational Diabetes Mellitus: Diagnostic Meta-Analysis

**DOI:** 10.1371/journal.pone.0102144

**Published:** 2014-07-11

**Authors:** Xudong Su, Zhaoyan Zhang, Xinye Qu, Yaqiang Tian, Guangzhen Zhang

**Affiliations:** 1 Department of Endocrinology, Liaocheng People’s Hospital, Liaocheng, China; 2 Department of Gynaecology and Obstetrics, Women and Children Hospital, Liaocheng, China; Weizmann Institute of Science, Israel

## Abstract

**Objective:**

To evaluate the accuracy of glycosylated hemoglobin A1c (HbA1c) for the diagnosis of postpartum abnormal glucose tolerance among women with gestational diabetes mellitus (GDM).

**Methods:**

After a systematic review of related studies, the sensitivity, specificity, positive likelihood ratio (PLR), negative likelihood ratio (NLR), diagnostic odds ratio (DOR), and other measures about the accuracy of HbA1c in the diagnosis of postpartum abnormal glucose tolerance were pooled using random-effects models. The summary receiver operating characteristic (SROC) curve was used to summarize the overall test performance.

**Results:**

Six studies met our inclusion criteria. The pooled results on SEN, SPE, PLR, NLR, and DOR were 0.36 (95% CI 0.23–0.52), 0.85 (95% CI 0.73–0.92), 2.4 (95% CI 1.6–3.6), 0.75 (95% CI 0.63–0.88) and 3 (95% CI 2–5). The area under the summary receiver operating characteristic (SROC) curve was 0.67 with a Q value of 0.63.

**Conclusions:**

Measurement of HbA1c alone is not a sensitive test to detect abnormal glucose tolerance in women with prior GDM.

## Introduction

Gestational diabetes mellitus (GDM) is described as any degree of glucose intolerance with on set or first recognition during pregnancy [1]. Up to 60% of women with a history of GDM will develop type2 diabetes mellitus (DM) within 5–10 years postpartum [2]. It is recommended that women with a history of GDM undergo a 2-hour oral glucose tolerance test (OGTT) within six months following pregnancy, and, if this is normal, assay of fasting plasma glucose or OGTT should be performed every three years [1]. Unfortunately, the rates of Postpartum evaluation are low [3]–[5]. HbA1c has been proposed as a diagnostic tool to identify people with undiagnosed diabetes, or who are at risk of diabetes [1]. HbA1c does not require fasting, it has better pre-analytical stability and reflects long-term glycaemic exposure better than current diagnostic tests based on fasting or post-load glucose measures. So using of HbA1c could improve postpartum testing rates for women with recent GDM. But the usefulness of HbA1c for the reassessment of carbohydrate metabolism status in postpartum women with a history of GDM remains controversial. The present meta-analysis was performed to establish the accuracy of HbA1c for diagnosis of postpartum abnormal glucose tolerance among women with gestational diabetes mellitus.

## Methods

### Search strategy and study selection

MEDLINE and EMBASE databases were searched, using the terms (postpartum.mp) AND (Gestational diabetes mellitus.mp OR GDM.mp) AND (hba1c.mp OR hemoglobin.mp) from 1985 to February 2014. We also reviewed the Cochrane Library to find relevant articles. We contacted experts in the specialty, and searched the reference lists from primary and reviewed articles. Although no language restrictions were imposed initially, for the full-text review and final analysis our resources only permitted the review of articles published in English. Conference abstracts and letters to the journal editors were excluded because of the limited data presented in them.

A study was included in the meta-analysis when it provided both the sensitivity (true-positive rate) and specificity (false-positive rate) of HbA1c for diagnosis of postpartum abnormal glucose tolerance among women with GDM, or when it provided HbA1c values in a dot-plot form, allowing test result to be extracted for individual study subject. Two reviewers (Z.Y.Z and X.D.S) independently judged study eligibility while screening the citations. Disagreements were resolved by consensus.

### Data extraction and quality assessment

Data extraction was carried out independently by two authors (Z.Y.Z and X.D.S). Disagreements were resolved by discussion between the two reviewing authors. Data retrieved from the reports included the author, publication year, participant characteristics, cutoff value, true-positive (TP), false-negative (FN), false-positive (FP), and true-negative data (TN).

The methodological quality of selected papers was evaluated using QUADAS-2, a tool for the quality assessment of studies of diagnostic accuracy [6]. This checklist consists of four key domains: patient selection, index test, reference standard and flow and timing. Within each study, the domains are assessed in terms of risk of bias and the first three of these domains are assessed in terms of concerns about applicability. Signalling questions as specified in the QUADAS-2 tool enable the reviewer to give each domain a rating of high, low or unclear.

### Statistical analysis

Standard methods recommended for meta-analyses of diagnostic test evaluations were used. The following measures of test accuracy were computed for each study: the sensitivity, specificity, positive likelihood ratio (PLR), negative likelihood ratio (NLR), and diagnostic odds ratio (DOR). The analysis was based on a summary receiver operating characteristic (SROC) curve [7]. The sensitivity and specificity for the single test threshold identified for each study were used to plot an SROC curve. A bivariate mixed effects model was used to calculate the average sensitivity, specificity, and the other measures across studies [8], and this analyses was performed using STATA version 12.0.

The heterogeneity of the results between studies was assessed statistically using the quantity *I^2^*, which describes the percentage of total variation across studies that is attributable to heterogeneity rather than chance [9]. Subgroup analyses was intended for investigation of operator experience and algorithm method however there was an insufficient number of studies. The relative DOR (RDOR) was calculated according to standard methods to analyze the change in diagnostic accuracy in the study per unit increase in the covariate. Since publication bias is of concern for meta-analyses of diagnostic studies, We tested for the potential presence of publication bias using Deeks funnel plots [10].

All analyses were undertaken using Review Manager 5.2 (The Cochrane Collaboration), Meta-DiSc statistical software 1.4 (Ramo’n y Cajal Hospital, Madrid, Spain) and STATA software, version 12.0 (Stata Corporation, College Station, TX).

## Results

### Study characteristics and quality

**Figure 1:**
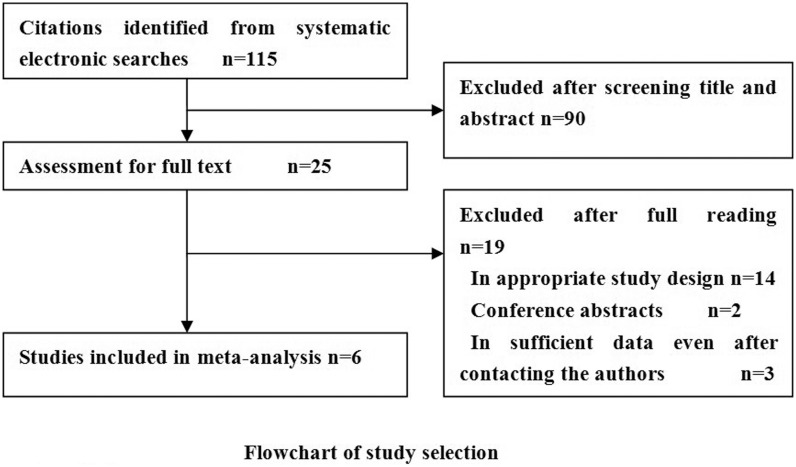
Flowchart of study selection and specific reasons for exclusion from the meta-analysis.

Figure 1 summaries the process of literature in identification and study selection. A total of 115 abstracts that met the inclusion criteria were retrieved. Two reviewers selected the relevant studies in dependently. After communicating with the authors, 6 reports offered sufficient data to build a two-by-two table and thus were included in the final meta-analysis [11]–[16]. The 6 included studies involving 435 patients with abnormal glucose tolerance (including impaired fasting glycemia or impaired glucose tolerance or type 2 diabetes) and 651 controls. All these studies were published from 2012 to 2013 and varied in sample size (from 54 to 364).

**Figure 2:**
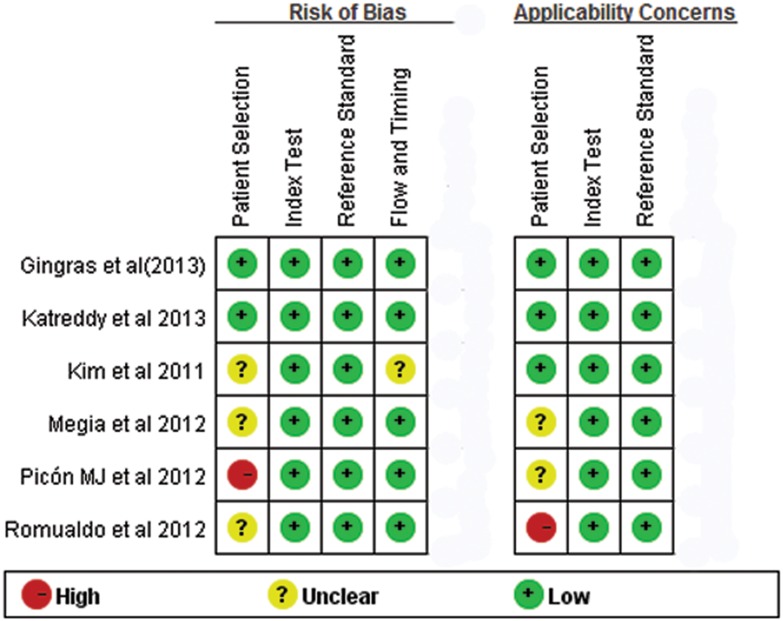
QUADAS-2 Risk of bias assessment.

In all studies, the Reference standard was 75 g OGTT and HbA1c was corrected in accordance with the recommendations of the National Glycohemoglobin Standardization Program (NGSP), based on the Diabetes Control and Complication Trial (DCCT) and the UK Prospective Diabetes Study (UKPDS). The clinical characteristics of these studies are outlined in Table 1. The results on the methodological quality of the included studies are presented in Figure 2.

**Table 1:** Summary of the studies included in the meta-analysis.

Author (year)	Ages (year)	Time after	Ethnic origin	Sample	Abnormalglucose	Reference	Cutoff (%)	TP	FP	FN	TN
		delivery		size	tolerancen (%)	standard					
Gingras et al (2013)	36.4±4.8	3.5±1.9 year	non-Hispanicwhite 94.6%	178	14 (79.2)	75gOGTT	5.7	68	6	73	31
Katreddy et al (2013)	29±4.6	6 weeks	Caucasians (70%)Asian 29.6%	203	32 (15.8)	75gOGTT	6.0	12	22	20	149
Picón et al (2012)	34.6±4.7	13.3±3.0 months	White 100%	231	106 (45.9)	75gOGTT	5.7	24	20	82	105
Romualdo et al (2012)	33.3±4.6	11±2 months	Caucasian 100%	56	32 (57.1)	75gOGTT	5.7	15	7	17	17
Megia et al (2012)	unknown	Within 1 year	European 91.5%,Arabic 5.5%	364	101 (27.8)	75gOGTT	5.7	14	7	87	256
Kim et al (2012)	36±4	18±12 months	White 73%, Asian 11%Afircan American 11%	54	23 (46.2)	75gOGTT	5.7	15	10	8	21

TP: true-positive; FN: false-negative; FP: false-positive; TN: true-negative; OGTT: oral glucose tolerance test.

### Diagnostic accuracy

**Figure 3:**
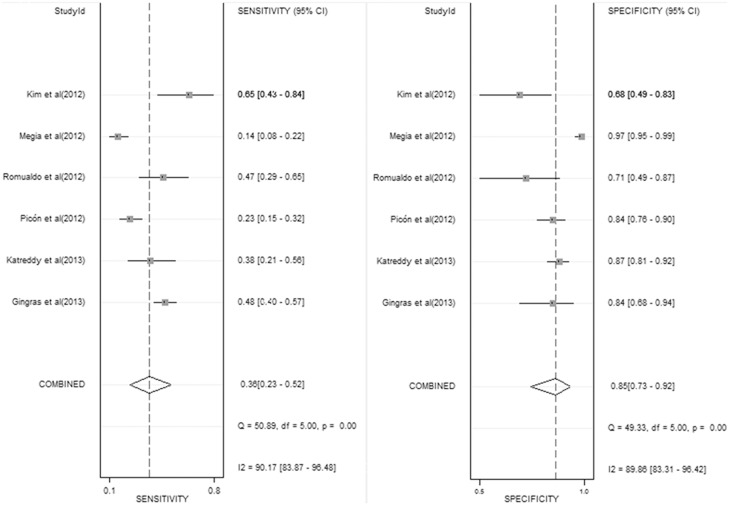
Forest plot of sensitivity and specificity for HbA1c in the diagnosis of postpartum abnormal glucose tolerance.

Figure 3 shows the forest plot of sensitivity and specificity for HbA1c in the diagnosis of postpartum abnormal glucose tolerance. The sensitivity ranged from 0.14 to 0.65 (0.36, 95% confidence interval [CI] 0.23–0.52), while the specificity ranged from 0.71 to 0.97 (0.85, 95% CI 0.73–0.92). The PLR was 2.4 (95% CI 1.6–3.6), the NLR was 0.75 (95% CI 0.63–0.88) and the DOR was 3 (95% CI 2–5). The *I^2^* value of all measures was 97%, indicating a significant heterogeneity across the included studies.

**Figure 4:**
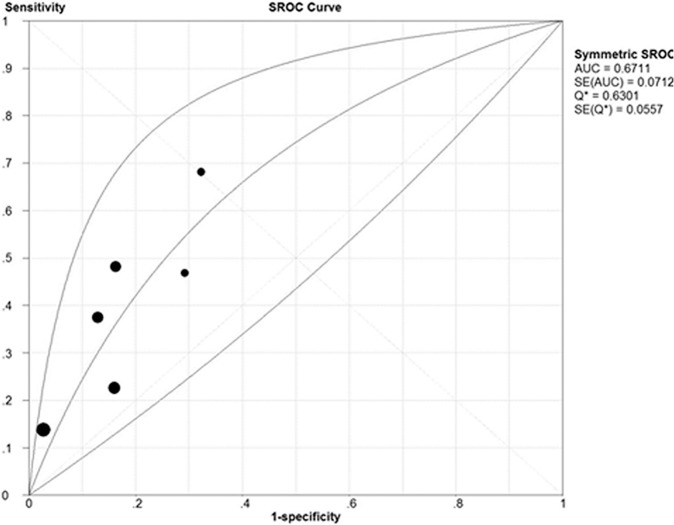
Summary receiver operating characteristic cure (SROC) for HbA1c. Each solid circle represents each study in the meta-analysis. AUC: area under curve.

The SROC cure presents a global summary of test performance, and shows the tradeoff between sensitivity and specificity. A graph of the SROC curve for the HbA1c showing ture-positive rates *vs* false-positive rates from individual studies is shown in Figure 4. The area under the curve (AUC) and an index Q value are discussed as useful summaries of the curve. Use of Q value as the summary measure assumes implicitly that false negative and false positive test results are of equal value. [17]. Our data showed that area under curve (AUC) was 0.67 (SE = 0.0712) with a Q value of 0.63 (SE = 0.0557), indicating a low level of accuracy.

Given the low number of studies included in the review, statistical subgroup analysis and covariate hierarchical modeling for investigation of heterogeneity were not performed due to low statistical power.

### Publication bias

**Figure 5:**
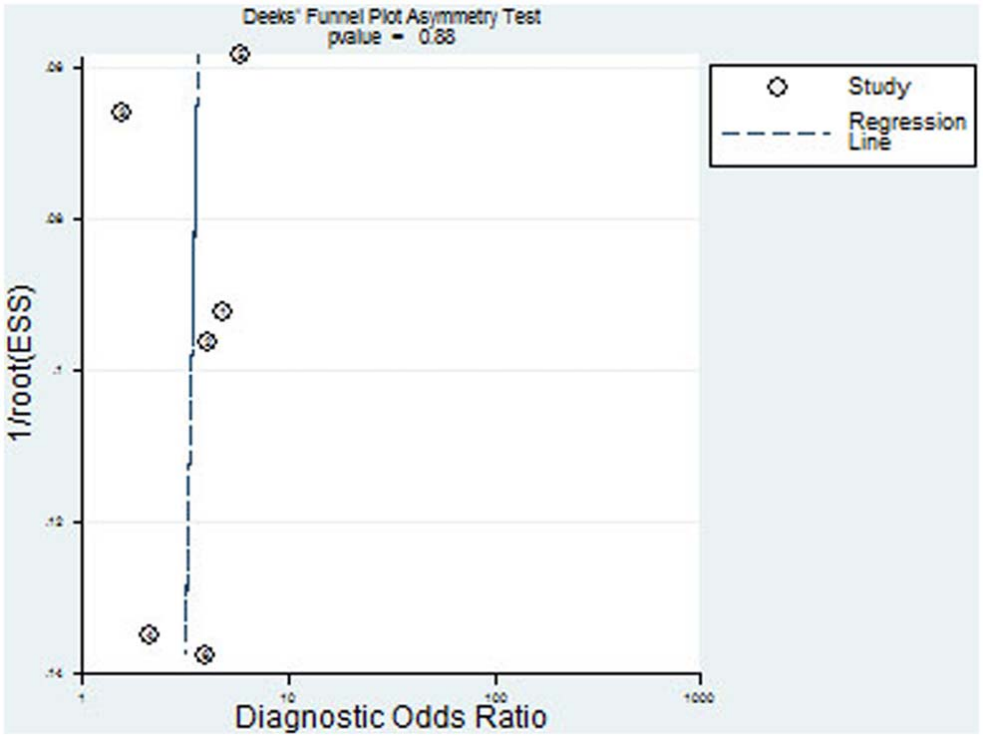
Deeks funnel plot asymmetry test of publication bias.

The Deeks fuunel plot asymmetry test showed insignificant publication bias (p = 0.88, Figure 5).

## Discussion

Postpartum testing is recommended for women with histories of GDM to diagnose diabetes and to stratify women for risk of future diabetes. The ADA [1] as well as the WHO consistently added HbA1c levels >6.5% (48 mmol/mol) to their diagnostic recommendations of overt diabetes. But the usefulness of HbA1c for the reassessment of carbohydrate metabolism status in postpartum women with a history of GDM remains controversial. To the best of our knowledge, this is the first meta-analysis designed to evaluate accuracy of HbA1c for diagnosis of postpartum abnormal glucose tolerance among women with gestational diabetes mellitus.

From this meta-analysis we can find that 40% (435/1086) of women with a history of GDM had abnormal glucose tolerance. Gingras et al [11] reported that the prevalence of abnormal glucose tolerance was 79.2% while Katreddy et al [12] reported only 15.8%. This difference may partly caused by the different test time after their most recent pregnancy.

The SROC curve is a technique for fitting a mathematical model to the scatter plot of sensitivity against (1-specificity). The area under the SROC curve (AUC) can summarize the inherent capacity of a test to discriminate the participant with disease from those without it. If a test has an AUC close to 1, it means that is a perfect test. The poor test usually has AUC close to 0.5. In our meta-analysis, we can find that the AUC was 0.67, it indicated that the HbA1c is not a suitable diagnostic tool for postpartum abnormal glucose tolerance.

The DOR is a single indicator of test accuracy that combines the data from sensitivity and specificity into a single number. The DOR is the ratio of the odds of positive test results in the patient with disease relative to the odds of positive test results in the patient without disease. The value of a DOR ranges from 0 to infinity, with higher values indicating the higher accuracy test performed. In the present meta-analysis, we found that the mean DOR was 3, also indicating a low level of overall accuracy.

The SROC curve and the DOR can’t explain the clinical situation, while the likelihood ratios are considered to be more clinically meaningful. A likelihood ratio describes how many times a participant with disease is more likely to receive a particular test result than those without disease [18]. In our present meta-analysis, a PLR of 2.4 suggests that patients with postpartum abnormal glucose tolerance with previous GDM have an approximately 2.4-fold higher chance of being HbA1c test-positive compared with patients with normal glucose tolerance. While the NLR was 0.75, all of these suggest HbA1c alone did not appear to be a sensitive test to detect pre-diabetes in women with prior GDM.

However, Gingras et al [11] found that the combination of HbA1c and waist circumference ≥88 cm had a higher sensitivity (79% vs. 48%) and a lower specificity (62% vs. 84%) for the ability to detect any glucose intolerance than HbA1C alone. Picón et al [13] also found the HbA1c test criterion had 22.64% sensitivity and 84% specificity to detect abnormal carbohydrate metabolism, but combination of HbA1c and fasting glucose ≥5.56 mmol/L had a higher sensitivity (83.02%) and a equal specificity. These studies suggested that the combination of HbA1c and other simple test could offer a sensitive test for the detection of abnormal carbohydrate metabolism in women with prior GDM.

An exploration of the reasons for heterogeneity rather than the computation of a single summary measure is an important goal of meta-analysis. In our meta-analysis, we found significant heterogeneity for sensitivity, specificity and NLR. The sources of heterogeneity might be different study population, ages and time after delivery. To assess the sources of heterogeneity in the studies, the meta-regression analysis was needed. However, only 6 studies included in the review, statistical subgroup analysis was not suitable due to the low statistical power.

The limitations of this meta-analysis are as follow. First, the exclusion of conference abstracts, letters to the editors and did not search for unpublished data, which probably caused publication bias. Second, because of the linguistic abilities of our study team, we only included English articles, and that might led to language bias. Third, HbA1c for diagnosis of postpartum abnormal glucose tolerance among women with prior GDM was concerned only in recent years. Our meta-analysis only included 6 studies and the sample size was small.

In conclusion, evidence suggests that HbA1c alone did not appear to be a sensitive test to detect abnormal glucose tolerance in women with prior GDM. The findings from our meta-anlysis should be confirmed in future research.

## Supporting Information

Checklist S1 **PRISMA Checklist.**
(DOC)
